# Harmonizing Identities: A Scoping Review on Voice and Communication Supports and Challenges for Autistic Trans and Gender Diverse Individuals

**DOI:** 10.1007/s10803-025-06768-1

**Published:** 2025-03-03

**Authors:** Bridgette Glanville, Jennifer Oates, Kitty-Rose Foley, Aida Hurem, Lily Osmetti, Kachina Allen

**Affiliations:** 1 Faculty of Health, Southern Cross University, Bilinga, QLD Australia; 2 School of Allied Health, Human Services and Sport, La Trobe University, Bundoora, VIC Australia; 3 National Centre of Excellence in Intellectual Disability Health, Mater Research Institute-University of Queensland, Brisbane, QLD Australia; 4 Queensland Centre of Excellence in Autism and Intellectual Disability Health, Mater Research Institute-University of Queensland, Brisbane, QLD Australia; 5 Faculty of Health, Southern Cross University, Lismore, QLD Australia; 6 Faculty of Education, Southern Cross University, Bilinga, QLD Australia

**Keywords:** Autism, Gender diverse, Wellbeing, Voice and communication, Transgender, Speech pathology

## Abstract

Autistic trans and gender diverse (TGD) individuals face unique voice and communication challenges compounded by minority stressors, impacting wellbeing and access to care. Speech pathologists are crucial in providing gender-affirming and neurodiversity-affirming support; however, guidance for working with this intersectional population remains limited. This scoping review mapped current knowledge on voice and communication challenges, identifies available supports, and demonstrates the limitations of existing guidance for speech pathologists. This review draws on 40 sources, including 29 peer-reviewed articles, 8 clinical guidelines, 2 books, and 1 position statement, identified through comprehensive searches of databases such as CINAHL, ERIC, Medline, APA PsycINFO, and grey literature in May 2024. Findings indicated that 96.8% of identified challenges pertained to communication, while fewer (3.2%) focused on voice-specific issues. Similarly, supports primarily addressed communication (91.3%), with 8.7% targeting voice needs. Communication challenges included barriers faced by autistic TGD individuals and those interacting with them, including healthcare professionals, peers, and family. Supports were identified as strategies and resources to enhance service delivery and client wellbeing, such as using visual aids or offering multiple communication options. However, significant gaps remain in addressing the unique voice and communication needs of this population, particularly regarding voice dysphoria, camouflaging versus identity disclosure, and communication within healthcare settings. Autistic TGD individuals represent a unique population whose needs are not sufficiently addressed by current guidance. This review highlights significant gaps in research and clinical practice and calls for improved clinical guidelines and specialized training for speech pathologists to enhance care.

**Supplementary Information:**

The online version contains supplementary material available at 10.1007/s10803-025-06768-1.

## Introduction

### The Autistic Community

Autism Spectrum Disorder (ASD) is a neurodevelopmental condition characterized by differences in typical social and communication behaviors, including challenges in social-emotional reciprocity, nonverbal communication, and understanding relationships (American Psychiatric Association, 2013). Additionally, autistic^1^ individuals may experience restrictive and repetitive patterns of behavior, interests, or activities that differ from those typically expected given their age or sociocultural context (World Health Organization, 2019). Communication differences can significantly impact the quality of life for autistic individuals, affecting their daily functioning, social relationships, and emotional wellbeing (Sturrock et al., 2022; Yau et al., 2023). It is the role of the speech pathologist to provide specialized communication supports that address these diverse communication needs, ensuring individuals receive appropriate care across the lifespan (Speech Pathology Australia, 2023).

The autistic community, navigating a predominantly ableist society, often experience autistic burnout (Raymaker et al., 2020) and minority stress (Botha & Frost, 2020), rendering them a vulnerable group. As a result, research has found that there is a concerningly high proportion of autistic individuals with mental health and wellbeing concerns including anxiety, depression and loneliness (Hossain et al., 2020; Schiltz et al., 2021). Further research has revealed a notable overlap between autism and trans and gender diverse (TGD) identities, with a higher proportion of autistic individuals identifying as TGD compared to the general population (Bouzy et al., 2023). In addition, autistic TGD individuals are likely to have greater mental health needs compared to transgender people who are not autistic, as well as cisgender and autistic peers (George & Stokes, 2018; Murphy et al., 2020). Consequently, health practitioners working at this intersection must understand and address the unique mental health needs, including gender dysphoria, which some individuals in this population experience (American Psychiatric Association, 2013).

### The Trans and Gender Diverse Community

The TGD community includes individuals whose gender identity does not align with the gender presumed for them at birth. Some TGD individuals experience gender dysphoria; distress often linked to a mismatch between an individual’s sex assigned at birth and their gender identity (American Psychiatric Association, 2013; World Health Organization, 2019). However, this distress is not solely internal; it is often compounded by external factors such as societal stigma and discrimination (Coleman et al., 2022). These challenges, shared with the autistic community, contribute to elevated rates of mental health concerns, including anxiety, depression, and suicidal ideation (Coleman et al., 2022). Such complexities must be carefully considered by all health practitioners, including speech pathologists, if they are to provide effective and affirming care.

TGD individuals may choose to engage in gender-affirming care to reduce gender dysphoria, increase gender euphoria, and combat the associated mental health crisis often related to minority stress (Dickey & Budge, 2020). Voice and communication training is an important support provided by available gender-affirming services. Speech pathologists are key in providing this care, helping clients modify their voice and communication style to better align with their gender identity (Coleman et al., 2022; Mills et al., 2017). For some TGD individuals, voice is a powerful means of expressing identity, and aligning one’s voice with their gender can significantly enhance wellbeing (Holmberg et al., 2023; Stewart et al., 2020).

However, providing gender-affirming voice and communication services extends beyond voice modification. Speech pathologists must also address social and psychological aspects of communication that affect wellbeing (Azul et al., 2022). Supporting a TGD client in overcoming the social and emotional challenges they face is part of the holistic care that speech pathologists can provide. This cannot be delegated solely to mental health professionals, as communication is closely tied to a person’s identity, emotional health and ability to self-advocate (Azul et al., 2022; Dodge et al., 2012; Strang et al., 2023). However, clear guidance and inclusive resources are needed for speech pathologists to be able to achieve this.

### Intersectionality of Autistic Trans and Gender Diverse Individuals’ Speaker Wellbeing

People who are both autistic and trans may present with more complex communication needs than those who identify as only trans or only autistic. For example, autistic individuals who are TGD are likely to communicate in highly individualized ways and express their gender related needs differently to those who are gender diverse and not autistic (Coleman et al., 2022).

Various countries have published national clinical guidelines for autism (Hyman et al., 2020; National Institute for Health and Care Excellence, 2012; Whaikaha Ministry of Disabled People & Ministry of Education, 2022; Whitehouse et al., 2018) and some have published guidance for working with TGD people (National LGBT Health Education Centre, 2020; NHS England, 2022; Oliphant et al., 2018; Telfer et al., 2018). However, none of these documents provide comprehensive national level guidance specifically for the intersection of autism and gender diversity. For example, in Australia, guidelines have been developed for the assessment and diagnosis of autism, as well as to support the wellbeing of autistic children and their families (Trembath et al., 2022; Whitehouse et al., 2018). However, no equivalent guidelines exist for the Australian context, that specifically guide supports for autistic adults or autistic TGD people. Comparatively, international comprehensive standards of care for TGD individuals across the lifespan exist, with some considerations incorporated for neurodivergent TGD individuals, although the latter are minimal (Coleman et al., 2022). As suggested by Bo et al. (2024), the best guidance that currently exists for the intersection of autism and gender diversity is the work from Strang et al. (2018) which describes some supports for social communication and voice. However, these guidelines are limited to group-style interventions for autistic TGD youth.

Thus, there is a need for additional research and guidelines that cover aspects of care that also consider the adult autistic TGD population and a wider range of service delivery models, particularly in Australia. Speech pathologists providing voice and communication services for this population must take proactive steps to build their skills and knowledge, not only in voice modification and communication, but also in supporting overall speaker wellbeing (Azul et al., 2022). However, this requires clear training and guidelines on how to safely and effectively provide gender-affirming and neurodiversity affirming services in tandem. Therefore, a scoping review was undertaken to comprehensively map the available literature, identifying the unique voice and communication challenges and supports available for this population. The research questions were:

What is the current body of knowledge regarding the voice and communication supports and challenges for the autistic trans and gender diverse community and what guidance currently exists for speech pathologists working at this intersection?

## Methods

### Design Protocol and Registration

This scoping review was developed in line with the Joanna Briggs Institute (JBI) guidance for scoping reviews (Peters et al., 2020). The review was also reported in accordance with the PRISMA extension for Scoping Reviews (PRISMA-ScR) (Tricco et al., 2018).

### Eligibility Criteria

Inclusion criteria were: (1) trans or gender diverse participants (either self-identified as TGD and/or medically diagnosed with gender dysphoria), (2) autistic spectrum conditions (either self-identified as autistic, medically diagnosed and/or those with autistic traits), and (3) focus on voice and/or communication. This also included sources that contained the above criteria but were written from the perspective of other individuals such as parents and professionals. For the latter, voice was conceptualized as strictly voice or vocal functioning issues or training, whereas communication was considered in its broadest sense to include social skills, as well as receptive and expressive language differences. Sources were excluded if they did not fulfill all three inclusion criteria or if the study did not have a primary focus on autism and/or gender diversity.

Only sources written in English were included. No inclusion restrictions were applied to the date of publication. However, the authors recognize that older research may include concepts and language that do not align with current neuro and gender-affirming standards. Consequently, a strengths-based approach was applied when interpreting the findings to avoid perpetuating outdated perspectives and to mitigate potential harm to the autistic and trans and gender diverse community. No limitations were placed on the age of the focus population. National and international peer reviewed papers, conference proceedings, and abstracts, as well as theses/dissertations and clinical guidelines were included. Other sources such as magazine and newspaper columns, social media posts or reports, websites, blogs, and fact sheets were excluded.

### Information Sources

A comprehensive literature search was conducted in May 2024, incorporating multiple strategies to ensure the inclusion of relevant sources. The search encompassed both medical and education-focused bibliographic databases to capture a wide range of pertinent studies. Databases searched included CINAHL, ERIC, Medline, and APA PsycINFO. The search terms used were:

- Autism OR Autistic OR ASD OR “autism spectrum” OR “autism spectrum disorder” OR asperger* OR neurodivergent OR “neuro divergent” OR neurodiverse OR “neuro diverse” OR “neurodiversity” OR “neuro diversity” OR neuro-diversity OR neuro-divergent
- **(AND)** transgender OR “gender diverse” OR transsexual OR transexual OR “gender variant” OR “gender non-conforming" OR non-binary OR nonbinary OR “non binary” OR “gender dysphoria” OR “gender queer” OR “gender fluid”
- **(AND)** “speech pathology” OR “speech-language pathology” OR “speech therapy” OR “speech therapist” OR “speech pathologist” OR “speech and language therapy” OR “voice and communication training” OR voice OR “voice training” OR “voice therapy” OR “voice and communication” OR “communication training” OR communication OR “communication supports” OR “communication challenges”

In addition, grey literature was explored via Google Scholar, and reference lists of relevant articles were manually reviewed to identify further eligible studies. An advanced Google search was also conducted to identify clinical guidelines, standards of care, and position statements that met the eligibility criteria, focusing on the first 100 results retrieved from the following countries where English is the primary language spoken and where identifying as transgender was not illegal: Australia, New Zealand, United States, United Kingdom, Canada, and Ireland. For this stage of the search, a simplified version of the above terms were used with the addition of “clinical guidelines”, “standard of care”, “position statements” and “position papers”.

### Search Strategy

The search strategy was initially developed by drawing on preliminary readings and discussions. It was subsequently reviewed and refined with input from an experienced university librarian. Given the anticipated scarcity of literature specifically addressing gender-affirming voice training for autistic individuals, the search concepts related to voice and communication were intentionally broad to encompass a wider range of terms.

### Selection of Sources of Evidence

The screening and selection of sources were managed using Covidence software. After automatic duplicate removal within Covidence, two authors independently screened titles and abstracts based on predefined eligibility criteria. Full-text articles selected for further review were then examined by another two authors. Discrepancies during title and abstract screening or full-text review were resolved independently by a separate author.

### Data Charting Process

A data extraction template was drafted in Microsoft Word and then transferred into Covidence. Data charting was completed by one author and later checked by a second author. Data were then exported from Covidence into a spreadsheet in Microsoft Excel for further analysis.

### Data Items

Data regarding source details (reference), study characteristics (study design) and participant information (location of participants, population description, participant age range) were extracted. Any data pertaining to the concepts of voice and/or communication in the context of autism and gender diversity were also extracted.

### Synthesis of Results

Descriptive statistics were used to describe source details, study characteristics, and participant information. Textual data related to the research questions, which focused on describing the current guidance for speech pathologists regarding the voice and communication challenges and supports for autistic TGD individuals, were synthesized and categorized using an inductive analysis approach such as described by Pollock et al. (2023).

## Results

### Selection of Sources of Evidence

**Fig. 1:**
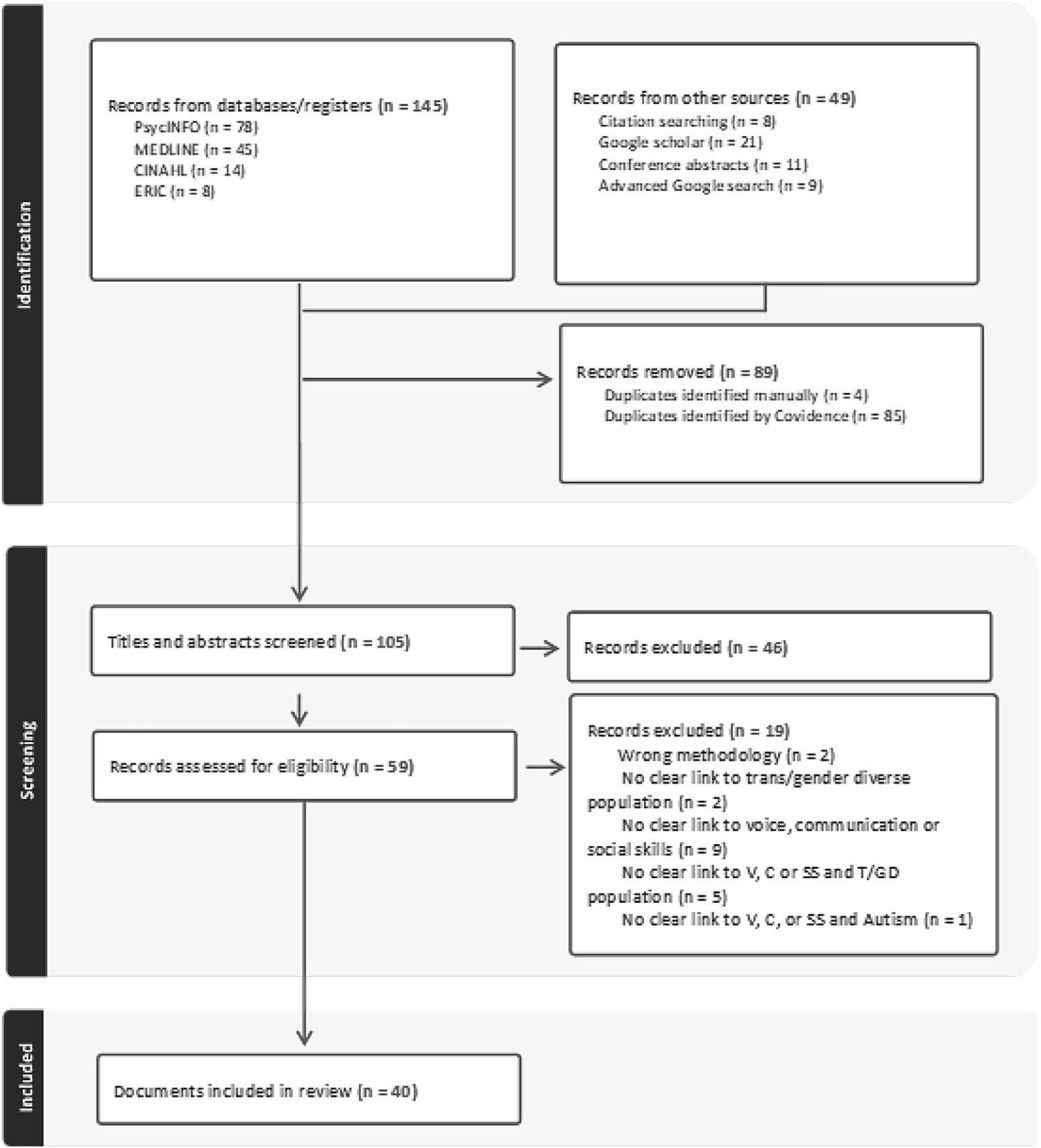
PRISMA flowchart: Source selection processes and reasons for exclusion

A search of 4 databases in addition to various grey literature searches yielded 145 sources which were uploaded to Covidence. After removing duplicates, 105 records were screened based on title and abstract. After excluding irrelevant sources, 59 full text sources were reviewed for eligibility resulting in the 40 documents included in this review. A flow chart depicting the details of this process is provided in Fig. 1.

### Characteristics of Sources of Evidence

A total of 40 sources of evidence (journal articles, guidelines, position statements and books) were included in this review as depicted in Fig. 1. Characteristics of the sources of evidence are presented within online resource 1 and synthesized descriptively below.

The data set was comprised of clinical guidelines or standards of care (*n* = 8), position statements (*n* = 1), books *(n* = 2), and peer reviewed articles (*n* = 29). As not all sources of evidence were journal articles, participant information was only extracted from relevant sources. Most studies focused on adolescents and adults (*n* = 10), followed by adults (*n* = 9), all ages (*n* = 6) and children and adolescents (*n* = 5). The majority of studies that reported a participant group included those who were autistic trans and gender diverse or had autistic traits and gender dysphoria. One study included participants that were mothers of autistic trans and gender diverse children (*n* = 1), while other papers contained participants who were professionals who work with autistic TGD individuals (*n* = 1) or were autistic TGD panel experts (*n* = 3). The location of participants was primarily from the United Kingdom (*n* = 9) and United States (*n* = 8).

### Synthesis of Results

**Fig. 2:**
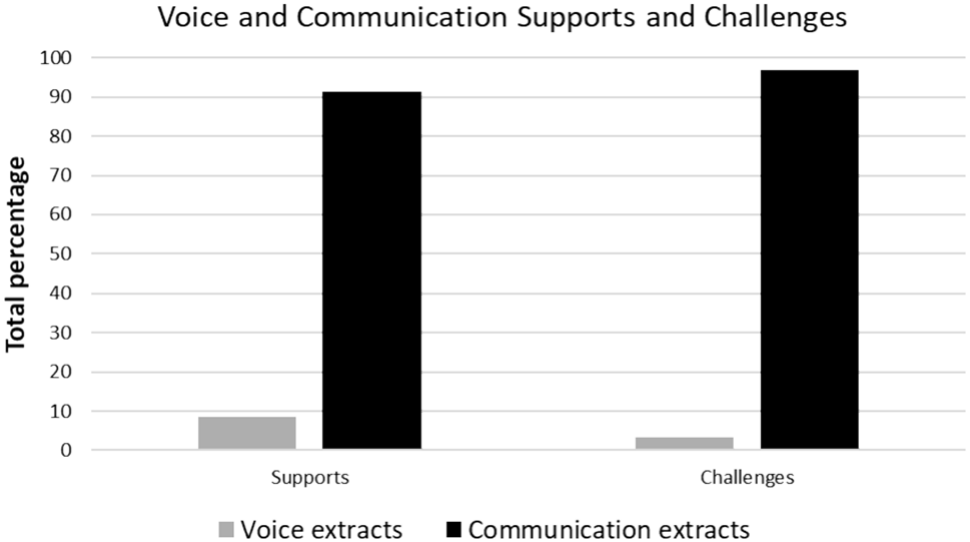
Voice and communication supports and challenges. Percentage of voice extracts compared to communication extracts from included sources

Out of a total of (*n* = 198) extracts pertaining to voice and communication, 95 were categorized as challenges, while 103 represented supports. Of the 95 challenges, 92 (96.8%) were related to communication broadly, with the remaining 3 (3.2%) specifically addressing voice-related issues (see Fig. 2). Similarly, of the 103 supports, 94 (91.3%) pertained to communication, while only 9 (8.7%) were voice-specific (see Fig. 2).

### Voice and Communication Supports

**Fig. 3:**
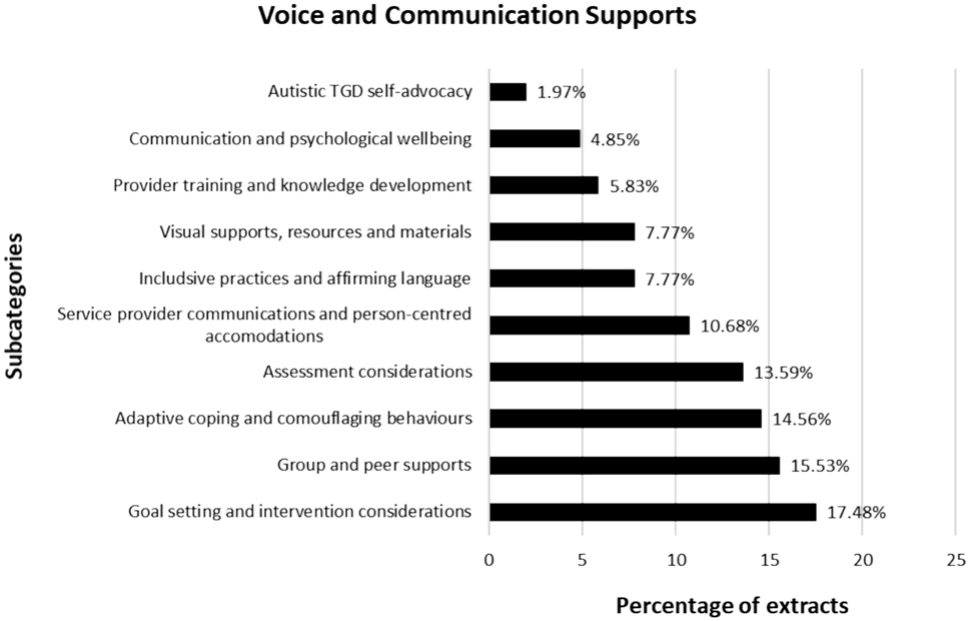
Voice and communication supports subcategories. Percentages of extracts relating to supports from included sources

Voice and communication supports were conceptualised as resources that can be used by or for autistic TGD individuals to assist them during social and communicative exchanges in a variety of contexts. From the 103 extracts related to supports, 3 broad categories were identified and these included (1) voice and communication supports for client wellbeing, (2) voice and communication supports in service delivery, (3) voice and communication supports for service provider professional development. From this, an additional 10 subcategories were formed as displayed in Fig. 3, along with their corresponding percentages. Further details surrounding these subcategories are described below supported by some example extracts from the data that best represented the subcategory.

### Category 1: Voice and Communication Supports for Client Wellbeing

#### Adaptive Coping and Camouflaging Behaviours

Adaptive coping and camouflaging behaviours (*n* = 15; 14.56%) included strategies used by autistic TGD individuals to navigate neurotypical social environments, often by adjusting their communication style to fit expected norms. Cook et al. (2022) also reported that participants modified their speech by slowing down or rephrasing for clarity, asking questions, and rehearsing phrases. These strategies often helped reduce anxiety and foster positive reactions. In fact, McQuaid et al. (2022) found that gender diverse adults reported elevated camouflaging on the Compensation subscale of the Camouflaging Autistic Traits Questionnaire (Hull et al., 2019) compared to cisgender adults. Compensation included the* “identification and close study of real-life or fictitious characters (e.g. from books, TV or other media) deemed to be socially adept. Through induction, autistic individuals may move from these exemplars to emulate patterns of social behavior and craft a persona that is gauged to have a high chance of social success”* (McQuaid et al., 2022, pp. 556–557).

#### Communication and Psychological Wellbeing

Some extracted data in the subcategory Communication and psychological wellbeing (*n* = 5; 4.85%), linked effective communication with improved emotional resilience and family support during gender transitions (Jacobs et al., 2014). Effective communication in this study enabled one adolescent participant to express their anxiety and gender incongruence which in turn enhanced their relationships, providing emotional relief and an increase in school performance. Similarly to allistic TGD people, supporting autistic TGD individuals to explore gender more flexibly was suggested as one way to alleviate distress and deconstruct the gender binary (Holt et al., 2016). However, it was noted that social transitioning can either be a source of anxiety, complicated by social differences, or an easy step in the transition journey (Bouzy et al., 2023).

### Category 2: Voice and Communication Supports in Service Delivery

#### Goal Setting and Intervention Considerations

Goal setting and intervention considerations (n = 18; 17.48%) was the largest category for service delivery supports, with a small number (5.3%) of these extracts related to guidance for speech pathologists on gender-affirming voice training. The voice-related elements focused on vocal health and education. For example, “*Psychoeducation regarding gender-related vocal parameters can be provided (i.e., intonation, volume modulation, oral resonance, and pitch), with an emphasis on vocal health (i.e., modifying vocal parameters in a healthy manner to prevent damage to vocal folds)” (*Strang et al., 2020*, p.13),* with many autistic TGD youth and some parents stating that advice on voice was wanted (Strang et al., 2021). Home practice for gender-affirming voice training was also encouraged to support generalization across settings (Strang et al., 2020). In terms of communication more broadly, sources stressed the importance of addressing not only general communication challenges but also autism related issues such as executive functioning, social communication, and self-awareness (Strang et al., 2018, 2020). Additionally, as recommended by Trembath et al. (2022), health care providers should support autistic children to work towards goals that support them to *“acquire skills that support their emerging autonomy, independence, self-identity (including gender identity), and capacity for self-advocacy*” (Trembath et al., 2022, p. 56). Social awareness and communication skills for navigating safe social interactions and transition to adulthood were also noted as key intervention areas (Genik, 2023; Jacobs et al., 2014).

#### Group and Peer Supports

Group and peer supports (n = 16; 15.53%) for autistic TGD youth provided vital opportunities for social communication and gender-related wellbeing. Online communities allowed these individuals to navigate social spaces without the challenges of face-to-face interactions (Kuvalanka et al., 2018). During face-to-face group interventions, consistent scripts and routines helped create welcoming environments for exploring diverse gender identities (Strang et al., 2020). While a multidisciplinary approach was discussed, speech pathologists were highlighted as key group leaders when supporting the voice, communication, and social language needs within these group settings (Strang et al., 2020). The development of peer relationships and shared vocabulary for autistic TGD individuals may also reduce the burden of having to over explain personal experiences during social interactions, thus leading to more meaningful connections (Peachey & Crane, 2024).

#### Assessment Considerations

Assessment considerations (*n* = 14; 13.59%) focused mostly on autistic TGD children and adolescents. Guidelines recommended that professionals assessing gender-diverse children consider developmental factors, neurocognitive functioning, and language skills (Coleman et al., 2022). Autism-specific guidelines, however, advised against universal screening for gender diversity during autism evaluations but suggested referrals to gender diverse services when co-occurrence was identified (Goodall et al., 2023). The Gender-Diversity and Autism Questionnaire (Strang et al., 2023) provided an autism friendly approach for gathering information. Clinicians were encouraged to use gender sensitive language, allowing clients to self-identify their preferred pronouns for clinicians to use (Goodall et al., 2023). For people who do not or cannot use verbal language to communicate, behavioural observation assessment approaches may be indicated (Gratton et al., 2023).

#### Visual Supports, Resources, and Materials

Visual supports, resources, and materials (*n* = 8; 7.77%) were found to aid discussions around gender identity, especially for autistic individuals. Visual tools such as the Genderbread Person (Killermann, 2018) were recommended (Bouzy et al., 2023). There was also a strong emphasis on making abstract gender concepts more concrete (Strang et al., 2020). For example, using concrete visual scales with autistic TGD youth to rate their perception of how their current voice does or does not align with their desired vocal presentation was recommended (Strang et al., 2020). These tools could then later support the identification of goals related to the modification of different vocal parameters (for example pitch, resonance, volume etc.). Other suggestions included checklists and flow charts, as well as explicit language, imagery and demonstrations (Coburn & Williams, 2023; Gratton, 2019; Strang et al., 2018, 2020).

#### Autistic TGD Self-Advocacy

The Autistic TGD self-advocacy subcategory (*n* = 2; 1.94%) highlighted that tools such as the Gender Diversity and Autism Questionnaire (Strang et al., 2023), designed with the language needs of autistic people in mind, may support self-advocacy in gender dysphoria. Peachey and Crane (2024) identified self-advocacy as being vital for autistic people to navigate gender related care. However, they also suggested that the responsibility should not solely fall on the individual themselves. This is particularly important given the cognitive and communication burdens they may face navigating multiple, complex health care systems.

### Category 3: Voice and Communication Supports for Service Provider Professional Development

#### Service Provider Communications and Person-Centred Accommodations

The subcategory Service provider communications and person-centred accommodations (*n* = 11; 10.68%) emphasised the need to offer more accessible methods of communication, such as phone calls, video calls, and emails, to improve client experiences and help clinicians manage caseloads efficiently (Bruce et al., 2023). Providers who listened and considered the individuals’ communication needs were found to positively impact the client experience (Bruce et al., 2023). Some sources suggested adapting communication by using clear and direct language, reducing idioms, asking clarifying questions, incorporating special interests, offering extended consultation times, and making changes to the clinic environment in order to improve service delivery (Cooper et al., 2023; Gratton, 2019; Rutter et al., 2023).

#### Inclusive Practice and Affirming Language

Inclusive practice and affirming language (*n* = 8; 7.77%) highlighted that healthcare providers could enhance support for autistic TGD individuals by addressing barriers to care through inclusive communication, accommodating diverse sensory and cognitive needs, and using language that respects the constancy or fluidity of gender (Genik, 2023; Gratton et al., 2023; Strang et al., 2021). Adapting forms, protocols, and communication methods to be more inclusive and meet the needs of autistic TGD individuals was also suggested (Gratton et al., 2023). Moreover, the *“use of universal or inclusive design practices in gender care settings could help to ensure that communication methods and care protocols support an effective informed consent process and attuned accommodations for navigating gender discernment and gender care”* (Gratton et al., 2023, p. 121).

#### Provider Training and Knowledge Development

The literature in the subcategory Provider training and knowledge development (*n* = 6; 5.83%), indicated that increased training on autistic experiences was essential for improving clinicians’ ability to communicate effectively and provide person-centered accommodations for autistic TGD individuals (Bruce et al., 2023). Training and professional development was also recommended by Coleman et al (2022) and Cooper et al. (2023), given the high prevalence of autism in TGD people. In addition, expanding understanding of narrative communication in autistic adults was reported as a way to reduce stereotypes and improve diagnostic accuracy (Coburn & Williams, 2023) which is particularly important for speech pathologists.

### Voice and Communication Challenges

**Fig. 4:**
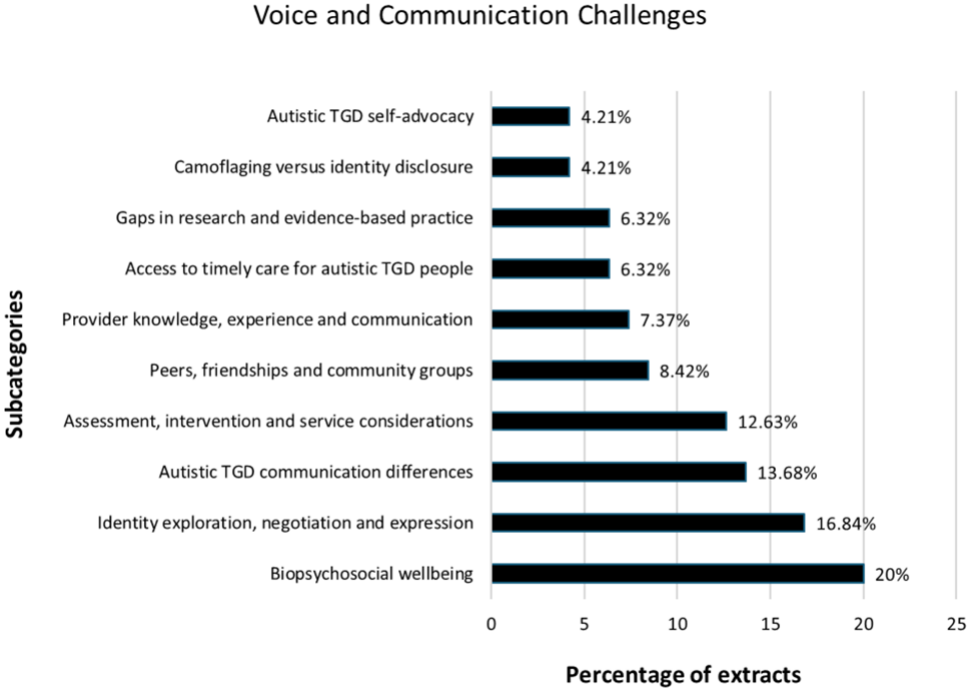
Voice and communication challenge subcategories. Percentages of extracts relating to challenges from included sources

Voice and communication challenges pertained broadly to communication barriers that exist for autistic TGD individuals or those engaging with them, including health and education professionals, family or peers. From the 95 extracts related to challenges, 3 broad categories were identified namely (1) voice and communication challenges for client wellbeing, (2) voice and communication challenges in service delivery, (3) voice and communication challenges for professional development. Of the 3 categories, a total of 10 subcategories were identified. These subcategories are displayed in Fig. 4, along with their corresponding percentages. Further details are provided below, with example extracts included for some subcategories.

### Category 1: Voice and Communication Challenges for Client Wellbeing

#### Biopsychosocial Wellbeing

Biopsychosocial wellbeing (*n* = 19; 20%) emerged as the most common voice and communication challenge for autistic TGD individuals. This subcategory aligns with the biopsychosocial model of health (Borrell-Carrió et al., 2004; Engel, 1977) which considers the interactions between an individual’s biology, their experiences and environments, and psychological factors such as self-esteem and wellbeing. This review revealed that due to communication differences, many autistic TGD individuals faced being dismissed by professionals often causing frustration (Bruce et al., 2023). They also expressed distress in varied ways (Cooper et al., 2023) and experienced heightened anxiety, particularly during social and medical transitions (Bouzy et al., 2023). Social isolation due to communication differences was common (Lehmann & Leavey, 2017). A lack of vocabulary to express identity had increased stress and contributed to poor self-esteem, especially among youth (Hillier et al., 2019). Employment challenges were also reported as a result of the inherent social skills required in many workplaces (Gratton, 2019), highlighting broader interpersonal and financial impacts on wellbeing. These individuals also faced heightened risks of harassment and violence, partly due to “*difficulty deciphering social cues, which made it harder for autistic gender-diverse individuals to read safe versus unsafe social situations*” (Strang et al., 2020, p. 15).

#### Identity Exploration, Negotiation and Expression

Identity exploration, negotiation, and expression (n = 16; 16.84%) was also a common voice and communication challenge. Participants in some studies struggled to articulate their gender identity due to a lack of vocabulary and limited opportunities for identity exploration (Hillier et al., 2019; Jacobs et al., 2014). Many had difficulty expressing a clear sense of self (Jacobs et al., 2014; Oliphant et al., 2018), often using language that felt mismatched with how others communicated about gender (Peachey & Crane, 2024). Cooper et al. (2023) noted that these challenges were exacerbated by social communication differences. Additionally, Peachey and Crane (2024) described that limited access to queer discourses further compounded these difficulties. The experience of navigating multiple identities for autistic TGD individuals was also reported as a challenge, adding further complexity to identity negotiation (Peachey & Crane, 2024). A common finding was that autistic TGD individuals often communicated differently about gender compared to non-autistic trans and gender diverse individuals, reflecting their “*varied interpretations of gender-related experiences given common differences in communication and thinking style*” (Coleman et al., 2022, p. 70).

#### Peers, Friendships and Community Groups

Peers, friendships, and community groups (n = 8; 8.42%) presented as a prominent challenge for some autistic TGD individuals, who often faced difficulties in social communication and forming relationships. Jacobs et al. (2014) highlighted that difficulty understanding different points of view and the emotional content in others speech contributed to social isolation and made it hard for one participant to connect with both neurotypical and LGBTQ+ peers. Fixed interests, while a strength and source of motivation for social engagement, may further complicate interactions (Strang et al., 2020) if they become all-consuming and do not align with the context or the interest of their peers. This may make it difficult for autistic TGD individuals to engage in both neurotypical and gender diverse spaces. Although some autistic TGD people desired friendships, maintaining them was often challenging (Hillier et al., 2019; Jacobs et al., 2014). This may leave them feeling disconnected or reluctant to join supportive communities due to the social skills required for participation (Jacobs et al., 2014; Peachey & Crane, 2024).

#### Camouflaging Versus Identity Disclosure

Camouflaging versus identity disclosure (n = 4; 4.21%) was the final subcategory identified as a challenge for wellbeing, especially mental health. As reported by Cook et al. (2022), some autistic TGD participants struggled with balancing camouflaging behaviors and self-disclosure. This was because “*disclosing personal information, responding to others, sharing opinions and using humour involve an element of social risk. Thus, if unsuccessfully deployed, they may increase the likelihood of negative evaluation*” (Cook et al., 2022, p. 415). Additionally, Cook et al. (2022) reported that some participants would use safety behaviors such as censoring speech or avoiding self-presentation practices that aimed to disclose their identity. However, these camouflaging behaviours often led to being perceived as anxious and less desirable socially.

### Category 2: Voice and Communication Challenges in Service Delivery

#### Autistic TGD Communication Differences

The subcategory Autistic TGD communication differences (n = 13; 13.68%) referred to the communication differences experienced by autistic TGD individuals. TGD individuals present with more autistic traits such as social and communication differences than their cisgendered peers (Jones et al., 2012; Nobili et al., 2017; Walsh et al., 2017). Other cognitive processes were also described by researchers in the field as being impaired alongside communication. For example, autistic TGD individuals may display “*features such as impaired ToM [theory of mind], an intolerance of ambiguity which could be interpreted as a manifestation of cognitive rigidity, a difficulty articulating their inner experience of gender, and persistent deficits in social communication and interaction*” (Jacobs et al., 2014, p. 278).

#### Assessment, Intervention and Service Considerations

Assessment, intervention, and service considerations (n = 12; 12.63%) was the largest subcategory identified for voice and communication challenges in service delivery. Many autistic TGD individuals were reported to face unique difficulties, as traditional gender-related assessments and interventions often did not account for autism-related communication differences (Gratton et al., 2023; Strang et al., 2018, 2023). Open-ended questions, requiring language formulation and broad conceptualisation, were especially challenging (Strang et al., 2023). Communication barriers extended to practical issues such as responding to appointment letters or making phone calls, further hindering access to care (Lehmann & Leavey, 2017). Diagnosing gender dysphoria was also reportedly complicated by difficulties with communication, self-awareness, and executive function (Strang et al., 2023). However,* “in line with WPATH guidance, treatment is contingent on an assessment of gender dysphoria, which depends on effective communication during consultations*” (Cooper et al., 2023, p. 40).

#### Access to Timely Care for Autistic TGD People

Access to timely care for autistic TGD people (n = 6; 6.32%) was found to be another clear challenge in service delivery which often resulted from communication barriers. Bouzy et al. (2023) highlighted that autistic children tend to begin gender-affirming care about 2.5 years later than neurotypical children, with delays potentially linked to communication differences in autism. Additionally, social differences may further impact access to timely and appropriate care (Lehmann & Leavey, 2017). Accessibility issues, including a lack of choice in communication, unmet sensory needs and misdiagnosis of mental health conditions, further hindered timely access to gender-affirming healthcare (Bruce et al., 2023)*.*

#### Autistic TGD Self-Advocacy

Autistic TGD self-advocacy (n = 4; 4.21%) was identified as a key challenge due to differences in thinking between autistic and neurotypical individuals (Strang et al., 2023) combined with systemic misunderstandings and doubt often stemming from communication differences and misconceptions about autism (Bouzy et al., 2023; Reframing Autism, n.d). For example, Coleman et al., (2022) reported that autistic TGD children may present with extra clinical complexities and therefore may find it difficult to self-advocate for their gender related needs.

### Category 3: Voice and Communication Challenges Professional Development

#### Provider Knowledge, Experience and Communication

Provider knowledge, experience, and communication (n = 7; 7.37%) emerged as a key challenge for autistic TGD individuals accessing gender-affirming healthcare. Some autistic TGD individuals described that most services have limited communication options which can lead to missed appointments or being discharged preemptively (Bruce et al., 2023). The experience of making and receiving phone calls was also a specific concern for autistic TGD individuals (Bruce et al., 2023; Gratton, 2019). Additionally, “*participants explained how they were unable to communicate effectively with professionals, due to the latter’s absence of knowledge around different communication and processing needs*” (Bruce et al., 2023, p. 199).

#### Gaps in Evidence-Based Research and Practice

Gaps in evidence-based research and practice (n = 6; 6.32%) highlighted the need for more inclusive studies on gender identity development. In particular, Bouzy et al. (2023) suggested further research involving individuals with intellectual disabilities or severe verbal communication disorders is needed. Many tools used to screen for autism in TGD individuals, such as the Social Responsiveness Scale (SRS; Constantino & Gruber, 2005), focus on communication and behavior and are deficit based. Communication accessibility issues in some tools, such as using complex language, were also likely to limit their effectiveness (Strang et al., 2023). Furthermore, additional research is required to deepen our understanding of identity development throughout the lifespan (Mo et al., 2024). This includes exploring the experience of camouflaging in autistic individuals, especially those who identify as gender diverse (McQuaid et al., 2022), as this may have important implications for speech pathologists in delivering effective social communication supports.

## Discussion

This scoping review mapped current evidence regarding voice and communication supports and challenges for the autistic TGD community. The findings from this review showed that the supports identified can be used to enhance client wellbeing, service delivery and the knowledge and skills of the professionals who support them, including speech pathologists. In particular, autistic TGD individuals expressed a need for more supportive communication options and clearer communication by professionals during and outside appointments to enhance access to care. They suggested that service providers should ask clients what their preferred communication method is regarding their care. This may include their preference for communication via phone calls or emails as well as explicitly discussing the details around the expected rate of communication (Bruce et al., 2023). Offering a range of person-centered accommodations speaks to the principles of universal design, a practice recommended by community and academic experts at the intersection of autism and gender diverse identities (Gratton et al., 2023). From the clinician’s perspective, health care providers have reported needing to adjust their practice and communication when working with autistic TGD individuals (Cooper et al., 2023). This includes offering extended consultation times due to perceived complexity, providing more structure to the clinic session, and using explicit language and concrete visual supports (Rutter et al., 2023; Strang et al., 2023). One of the visual resources included in this review was the GenderBread person (Killermann, 2018), a free resource that was initially published in 2011 by a social justice advocate. Similar resources have since been developed by and for the TGD community, such as the Gender Unicorn (Trans Student Educational Resource, 2015), which are considered to be more trans inclusive.

However, this review has also provided clear evidence that there are unique communication challenges for autistic TGD individuals and those engaging with them, that may contribute to poor outcomes and negatively impact care. Current guidelines in gender-affirming care recognize that autistic TGD individuals communicate in highly individualized ways and may face difficulties advocating for their gender-related needs (Coleman et al., 2022). This review further highlighted that autistic TGD individuals often exhibit subtle but significant differences in communication compared to their neurotypical counterparts (Tollit et al., 2024). Visual resources such as the GenderBread Person and the Gender Unicorn, while widely used in the TGD space, rely on abstract and metaphorical representations, requiring individuals to conceptualize gender in ways that conflict with the explicit, concrete communication preferences of autistic individuals. This disconnect underscores a critical gap in existing resources and care practices for autistic TGD specifically.

Autistic TGD individuals also report having a lack of vocabulary for how they identify and experience gender, in part due to differences in communication and limited access to community representation (Hillier et al., 2019; Peachey & Crane, 2024; Strang et al., 2018). Some autistic TGD individuals may desire peer and romantic relationships but experience difficulties connecting with others due to social communication differences (Hillier et al., 2019). Additionally, some individuals may face challenges in negotiating their gender identity due to the complexity of holding multiple intersecting stigmatized identities and articulating this experience. This can create difficulties in developing and explaining a cohesive self-concept which may also influence their ability to engage fully with the queer community (Peachey & Crane, 2024).

Camouflaging, a coping behavior frequently employed by autistic individuals in social settings with allistic people, has been shown to have adverse effects on well-being, with some individuals reporting it as emotionally and cognitively taxing (Cook et al., 2022; Hull et al., 2017). Nevertheless, this review highlights that certain autistic individuals, including those who are gender diverse, employ camouflaging as a strategic means of communication to facilitate social engagement and form friendships with neurotypical peers (Cook et al., 2022; Hull et al., 2017). However, a notable gap in the literature is the nuance around how autistic TGD individuals experience concealment or identity disclosure related to their gender and how this intersects with their experience when masking autistic traits. Research in this area would be particularly valuable for practitioners, such as speech pathologists, who work at the intersection of autism and gender diversity and aim to deliver services that support social communication in a way that is both neuro-affirming and gender-affirming.

More broadly, the process for health care providers attempting to establish a diagnosis of gender dysphoria or seeking to understand an autistic individual’s sense of gender is complex due to communication differences (Strang et al., 2018). Traditional interviews may rely on open-ended questions that may be more difficult for autistic people (Strang et al., 2023). Some autistic individuals do not necessarily use speech as their primary mode of communication and instead may use alternative and augmentative communication (AAC) systems. Therefore, clinical efforts to characterize gender based on communication and responses to verbal queries may prove ineffective (Gratton et al., 2023), particularly if the individual does not have access to the icons, vocabulary or synthetic-voice options that best matches that of their gender identity. However, in line with WPATH guidance, treatment is contingent on an assessment of gender dysphoria, which depends on effective communication during consultations (Coleman et al., 2022; Cooper et al., 2023). This places speech pathologists and other healthcare providers who may lack the knowledge and skills to address the unique communication needs of autistic TGD individuals at a stalemate, potentially delaying or limiting access to care.

As described above, the consequences of these voice and communication challenges impact a range of stakeholders. Most concerning is the impact on the overall wellbeing of autistic TGD individuals. It is the role of a speech pathologist to address an individual’s voice and communication needs (Speech Pathology Australia, 2020), as well as to support speaker wellbeing for all people (Azul et al., 2022; Speech Pathology Australia, 2018). Yet this review exposes that there is limited support available to professionals such as speech pathologists that directly addresses speaker wellbeing in autistic TGD individuals. In fact, the ‘Biopsychosocial wellbeing’ challenges subcategory represented 20% of the data, while the ‘Communication and psychological wellbeing’ supports subcategory represented only 4.85%. This suggests that there is a significant lack of guidance available to adequately support the communication needs of autistic TGD people and enhance speaker wellbeing. Findings from this review also show that voice dysphoria is more prevalent among individuals with autistic traits than those without (Tollit, 2022). However, results also indicate that there is very little understanding of the voice specific challenges and supports for autistic TGD individuals. This suggests that speech pathologists are unlikely to know how best to meet the unique voice needs of this population.

Current guidance often advises referring individuals to specialist gender or autism services when one service lacks expertise or training in addressing the needs of the other (Coleman et al., 2022; Whitehouse et al., 2018). This approach highlights a gap in integrated care for individuals who require support in both areas. Voice and communication are closely intertwined and integral to identity expression for autistic TGD individuals, making it insufficient for speech pathologists to routinely refer clients to other clinicians with more expertise in either area. Ethically, referring autistic TGD individuals to separate autism or gender-specialized services raises practical concerns that risk negatively impacting client well-being. For autistic clients who experience heightened distress in response to change, transitions between services may present as an added risk of unnecessary stress, potentially contributing further to autistic burnout (Raymaker et al., 2020). TGD individuals often face significant wait times for gender-affirming care (Chaplyn et al., 2023; Strang et al., 2018). This in turn may add further to the delay autistic TGD individuals often experience in receiving timely and appropriate care, as identified within this review (Bouzy et al., 2023; Bruce et al., 2023; Gratton, 2019; Lehmann & Leavey, 2017). For example, the unique communication and cognitive differences in autism can delay critical diagnoses and access to gender-affirming care. This may result from difficulties in expressing feelings of gender incongruence and a lack of understanding from others (Bouzy et al., 2023; Strang et al., 2018). Concerningly, this review also revealed that autistic TGD individuals report receiving incorrect diagnoses, with communication differences leading to diagnoses of other mental health conditions, further delaying access to essential care (Bruce et al., 2023). Hence, more speech pathologists and other health professionals with the knowledge and skills to work with autistic TGD people are needed to ensure equitable and timely care.

The clinical guidance found in this review also recommends that health care providers working with TGD people consider the developmental factors, neurocognitive differences and language skills that may be seen in those with autism (Coleman et al., 2022). However, this suggestion was specifically focused on pediatric populations and doesn’t clearly describe how professionals should navigate these complex considerations in clinical practice. It also presumes that clinicians, such as speech pathologists, possess the necessary confidence and competence to work with autistic individuals. Previous research by Plumb and Plexico (2013), however, has revealed that many speech pathologists feel that they would have benefited from more experience and training with autistic children to enhance their knowledge and confidence. Additionally, the focus of guidance surrounding autistic TGD children highlights that there remains a critical gap in addressing the unique voice and communication needs of autistic TGD adolescents and adults. This is consistent with a recent study by Bo et al. (2024) which emphasized the need for more research and clinical guidance that prioritizes improved outcomes for autistic TGD people navigating the dual dimensions of autism and gender diversity using a developmental, lifespan and strengths-based approach.

Thus, findings from this review in combination with the study reported by Bo et al. (2024) underscores the urgent need for further research with this community, and specialized clinical guidelines and training for speech pathologists operating at this intersection. While the findings from this review add to the foundational knowledge required to inform such guidelines, further research is needed, particularly around autistic TGD speaker wellbeing. However, to create effective guidelines and training, it is essential to also engage both speech pathologists and community members with lived experience at this intersection. Thus, while this review contributes initial insights, further research and collaboration with clinicians and autistic TGD individuals will be crucial in developing a comprehensive, evidence and strengths-based framework that fosters ethical, inclusive, and holistic care for this population.

## Limitations

This review highlights several limitations and areas for future research. Current clinical guidelines for the autistic community and the TGD community, have been broadly applied to autistic TGD individuals. While some practices outlined within these guidelines may be beneficial, the generalisation of this guidance has the potential to overlook the unique voice and communication needs of this intersectional population. Many of the voice and communication supports identified within this review are derived from studies that have not specifically evaluated their effectiveness for the autistic TGD population. Furthermore, there remains a significant gap in research involving autistic TGD individuals and their families to explore their voice and communication needs, preferences and barriers in accessing care. This includes understanding the experiences of those who use high-tech, low-tech and no-tech AAC options, as well as those who choose not to access voice and communication services. Finally, the literature lacks input from speech pathologists. Despite higher reported levels of voice dysphoria in autistic individuals who are gender-diverse, little is known about how speech pathologists address these challenges and promote gender euphoria. Their perspectives are crucial for addressing the unique challenges of working with autistic TGD clients and supporting clients to achieve positive experiences with their voice. Therefore, research should also investigate speech pathologists’ experiences, including their perceived education, training and confidence levels when working with this population. Interviews, surveys and focus groups could be useful in advancing knowledge in these areas.

## Conclusions

This scoping review highlights the critical need for enhanced voice and communication supports tailored to the unique needs of the autistic TGD community. Autistic TGD individuals face unique communication challenges. These challenges, in turn, impact speaker wellbeing. While some existing supports show potential to improve speaker wellbeing and enhance clinical practice, the findings demonstrate significant gaps in both research and guidance for professionals, including speech pathologists. Current clinical guidelines often emphasize referral to autism or gender specialists, yet this approach may unintentionally delay care and overlook the capacity of professionals already engaged with autistic TGD clients, to provide necessary services. However, in order to build the capacity of the workforce, such as within the field of speech pathology, the development of specialized training and comprehensive clinical guidelines to deliver neuro-affirming and gender-affirming care for the autistic TGD community is strongly recommended. Further research with this population is needed to deepen understanding of their voice and communication needs, with a particular focus on enhancing speaker wellbeing. Perspectives from speech pathologists would also prove valuable to support the development of training specific to the needs of this discipline when working with autistic TGD people.

## Supplementary Information

Online resource 1: Source characteristics—supplementary materials.

Below is the link to the electronic supplementary material.

Supplementary file1 (XLSX 18 KB)

## Footnotes

1 Many different language and terminology preferences exist when referring to the autistic and autism community. Ultimately, in research and clinical practice, one is encouraged to follow the preferences of the autistic individual themself and how they would like to be referred to regarding their autism. For this research, identity-first language such as autistic, autistic individual/s and autistic people/person will be used. This is consistent with the language used by Australia’s national source of evidence for best practice in relation to autism across the lifespan and the spectrum, the Autism Cooperative Research Centre (CRC) (Autism CRC, n.d.).
